# The association between high jugular bulb and mastoid pneumatization in adults

**DOI:** 10.3389/fneur.2023.1331604

**Published:** 2024-01-08

**Authors:** Chenyu Chen, Simin Weng, Zhifeng Chen, Yuqing Chen, Guangnan Yao, Xiying Huang, Xi Gu, Chang Lin

**Affiliations:** ^1^Department of Otorhinolaryngology, Head and Neck Surgery, The First Affiliated Hospital of Fujian Medical University, Fuzhou, China; ^2^Department of Otorhinolaryngology, Head and Neck Surgery, National Regional Medical Center, Binhai Campus of the First Affiliated Hospital, Fujian Medical University, Fuzhou, China; ^3^Fujian Provincial Clinical Medical Research Center for Ear, Nose and Throat Difficulty Diseases, Fuzhou, China; ^4^Fujian Branch of National Clinical Medical Research Center for Otorhinolaryngologic Diseases, Fuzhou, China; ^5^Fujian Institute of Otorhinolaryngology, The First Affiliated Hospital, Fujian Medical University, Fuzhou, China

**Keywords:** high jugular bulb, mastoid pneumatization, high resolution computed tomography, temporal bone, 3D

## Abstract

**Purpose:**

The purpose of this study was to analyze the relationship between the degree of high jugular bulb (HJB) and mastoid pneumatization using high-resolution computed tomography (HRCT).

**Methods:**

Between April 2019 and June 2022, HRCT of the temporal bone was retrospectively analyzed in 1,025 patients. By excluding the other coexistent pathologies, 113 patients with HJBs were recruited for the study. The degree of the HJBs were defined as follows: Grade I, JB situated between inferior annulus of tympanic membrane and cochlear basal turn (CBT). Grade II, JB situated between CBT and lateral semicircular canal (LSC). Grade III, JB situated above LSC. The volume of mastoid pneumatization was based on HRCT images using a 3D reconstruction.

**Results:**

There were 32 male and 81 female subjects (mean age, 41.2 ± 14.0 years; age range, 18–80 years). The male group included 16 Grade I, 28 Grade II and 6 Group III HJB subjects. The female group included 38 Grade I, 62 Grade II and 31 Group III HJB cases. In the different groups of HJB, the mastoid cell volume differences were also not statistically significant (*p* = 0.165). In the classification, Grade II was most common (90/181, 49.7%).

**Conclusion:**

This study found no correlation between mastoid air cell volume and HJB, suggesting that HJB may not affect the mastoid air cell development and disease occurrence. These data must be considered exploratory, requiring more extensive cross-sectional studies.

## Introduction

The mastoid pneumatization is thought to play an important role in middle ear physiology. It acts as an air reservoir and pressure regulator, protecting the delicate inner ear structure from changes in ambient temperature and air pressure (1). Various studies have aimed to investigate the correlation between middle ear disease and the mastoid air cell system. Hypopneumatized mastoid system has been shown to be a risk factor for the development of various middle ear diseases (2). To our best knowledge, high jugular bulb (HJB) is a congenital variant that has been shown to affect middle and inner ear structures. An abnormal jugular bulb (JB) can even erode into the facial nerve, vestibular aqueduct, posterior semicircular canal and cause corresponding clinical symptoms, such as pulsatile tinnitus, conductive hearing loss, or vertigo (3–5). As previously reported, pneumatization of the mastoid is one of several factors that have been hypothesized to influence the variabilities and variations of these vessels (6). While, there is still a controversy on the relationship between the degree of JB and the degree of mastoid pneumatization. Graham (7) and Aladeyelu et al. (6) proposed that the height and shape of the JB were related to pneumatization of the temporal bone. However, Orr and Tod (8) and Dai et al. (9) showed that JB position and shape were unrelated to the mastoid pneumatization. Few literatures have quantitatively analyzed the morphological and positional relationship between the degree of HJB and mastoid pneumatization. The purpose of this study was to elucidate the role of HJB in the total volume of the mastoid air cell system, provide more detailed information, and provide a reference for surgical intervention and disease prevention.

The mastoid ventilation system is one of the most significant aeration systems in the body (10). The mastoid air cell system is fully developed at approximately 15 years old for males and 10 years old for female (11). The mastoid air cell system acts as an air reservoir for the middle ear, but the physiologic functions of the mastoid air cell system is poorly understood. One hypothesis is that the degree of mastoid pneumatization is hereditary (11). Another hypothesis is that the condition of the middle ear cavity affects the degree of mastoid pneumatization (12). Factors such as chronic otitis media, age, race, genetic factors, and environmental conditions are considered to be the main factors in the development of the mastoid air cell system. The development and general characteristics of mastoid air cell system are well documented in the literature (11). The pneumatization of the mastoid region may be divided into three parts: sclerotic, diploic and pneumatic mastoid. When the mastoid process lacked with air cells was called sclerotic mastoid. Diploic mastoid was less dense than sclerotic ones due to the presence of marrow spaces. Pneumatization refers to the process by which mastoid air cells develop within the mastoid process. In this study, three-dimensional volume was used to quantify the degree of mastoid pneumatization which may be closer to the real situation.

In general, the vascularity of the temporal bone varies greatly among individuals and between right and left ears (13). As reported, the HJBs are not rare in population (4). This developmental temporal bone abnormality affects the function of the middle ear and inner ear, and may be part of the pathophysiology of the ear. At the same time, the exact mechanism and factors affecting the pneumatization of mastoid cells are discussed. Both HJB and mastoid pneumatization can affect the development of the inner and middle ear. Therefore, examining the correlation between these two aspects has positive implications for a better understanding of developmental processes and associated disease progression. Few literatures have quantitatively analyzed the morphological and positional relationship between the degree of HJB and mastoid pneumatization. Based on the digital HRCT images of temporal bone, the present study was undertaken to examine the relationship between the HJB and the mastoid pneumatization.

## Methods

### Subjects

Between April 2019 and June 2022, temporal bone high-resolution computed tomography (HRCT) of 1,025 patients were retrospectively analyzed. One hundred thirteen consecutive patients (32 male and 81 female subjects; mean age, 41.2 ± 14.0 years; range, 18–80 years) with HJBs were included in the study. Nine hundred twelve subjects with additional coexistent pathologies such as nasal septal deviation, nasal polyps, rhinosinusitis, tumors, tympanosclerosis, atelectasis, otitis media, nasopharyngeal lesions and previous surgery were excluded. All HRCT scans were obtained at 120.0 kV, 440 mA, and the slice thickness was at least 0.5 mm. The axial cuts were obtained parallel to the orbito-meatal baseline and viewed in the standard settings.

### Analysis of preoperative HRCT scans

**Figure 1:**
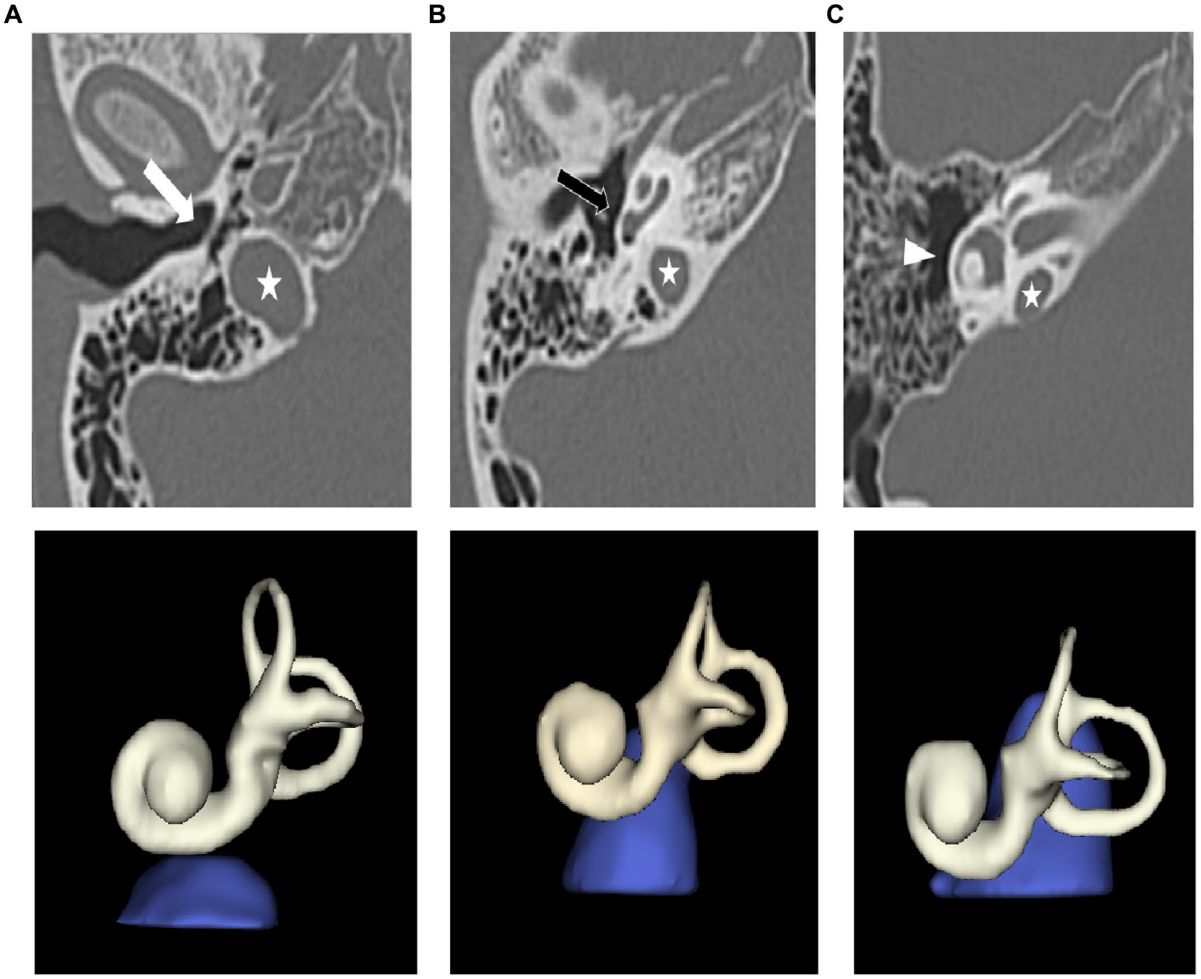
Three different degree of HJB. Grade I: JB situated between inferior annulus of TM and CBT **(A)**. Grade II: JB was between CBT and LSC **(B)**. Grade III: JB was above LSC **(C)**. Jugular bulb (JB, asterisk), tympanic membrane (TM, white arrow), cochlear basal turn (CBT, black arrow), lateral semicircular canal (LSC, triangle). The corresponding 3D reconstruction of three different degree of HJB are below the respective plane images.

**Figure 2:**
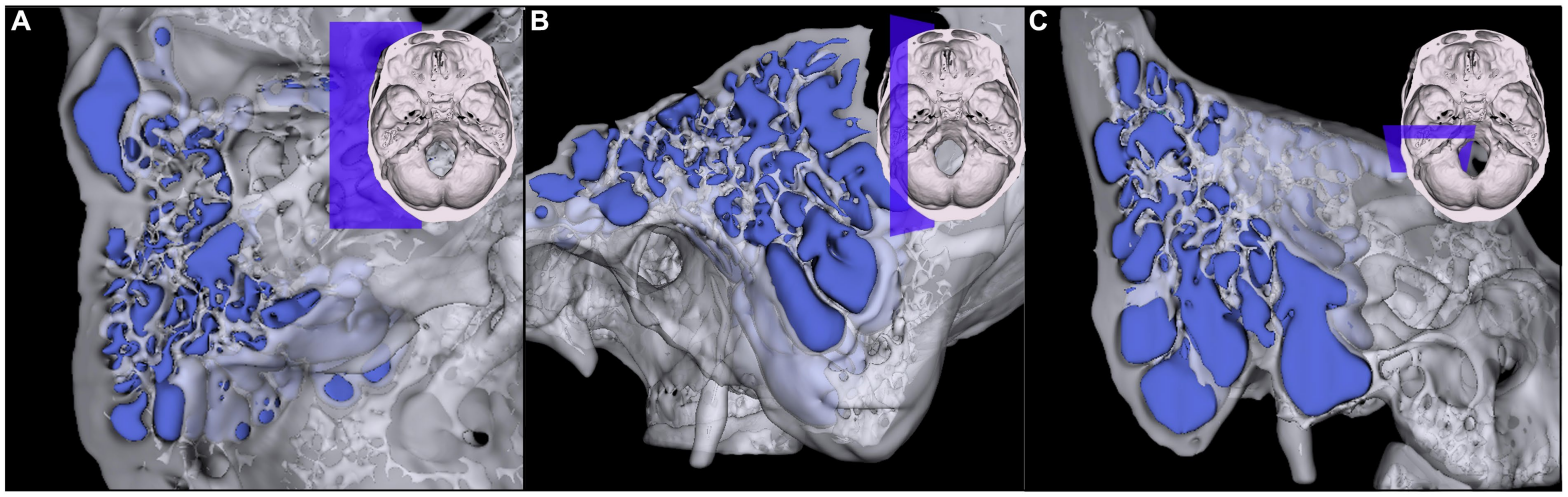
Three-dimensional reconstruction of different positions of mastoid air cells. **(A)** horizontal plane, **(B)** sagittal plane, **(C)** coronal plane.

Image J^1^ and 3D Slicer imaging software (version 4.10.2, Boston, United States) were used to review and identify HRCT images. The three adjacent structures, the inferior annulus of tympanic membrane, the cochlear basal turn (CBT) and the lateral semicircular canal (LSC) were used as classification criteria because they were easily visible. The HJBs were then divided into the following three groups: Grade I, JB situated between inferior annulus of tympanic membrane and CBT. Grade II, JB was between CBT and LSC. Grade III, JB was above LSC (Figure 1). Radiological measurements were performed independently blinded by two senior radiologists and any differences in opinions were resolved by consensus. The grading standard was selected from previous literature as proposed by Woo et al. (14). In this study, a Hounsfield unit (HU) segmentation threshold from −1,024 to −200 was used to represent the area of the mastoid air cells (15). By summing all volumes of each slice, the total volumes of mastoid pneumatization were automatically calculated by 3D reconstruction expressed in cubic centimeters (Figure 2). It was also important to note that the area measured in the present study did not include the tympanic cavity or the portion of the petrous apex.

### Statistical analysis

All results were statistically analyzed using SPSS for Windows 26.0 (Chicago, IL, United States) and *p* < 0.05 was considered significant. Parameters were expressed as mean ± standard deviation. Differences between HJB groups and mastoid air cell volumes were assessed using one-way analysis of variance (ANOVA) and the post hoc Bonferroni test. Pearson correlation coefficients were calculated to evaluate the association between the degree of HJBs and mastoid pneumatization. The qualitative incidence of mastoid pneumatization in patients with the severity of HJBs were performed using the Pearson chi-square test.

## Results

A total of 113 patients consisting of 102 right-sided and 79 left-sided HJBs were included in the study. It consisted of bilateral HJBs (68 ears) and unilateral HJB (45 ears; left:34, right:11). There were 32 male and 81 female patients whose average age was 41.2 ± 14.0 years, ranging from 18 to 80 years. The male group included 16 Grade I, 28 Grade II and 6 Group III HJB subjects. The female group included 38 Grade I, 62 Grade II and 31 Group III HJB cases (Table 1).

**Table 1:** Comparison of the mastoid air cell volume with different degree of high jugular bulb in two sexes.

	Male		Female		Volume
No HJB	14	6.54 ± 2.93	31	3.47 ± 1.90	4.43 ± 2.66
I	16	5.08 ± 3.24	38	4.19 ± 2.31	4.46 ± 2.62
II	28	5.95 ± 3.05	62	4.14 ± 1.97	4.70 ± 2.49
III	6	6.10 ± 2.66	31	3.11 ± 1.70	3.59 ± 2.15
*p*-value		0.617		0.055	0.165

**Figure 3:**
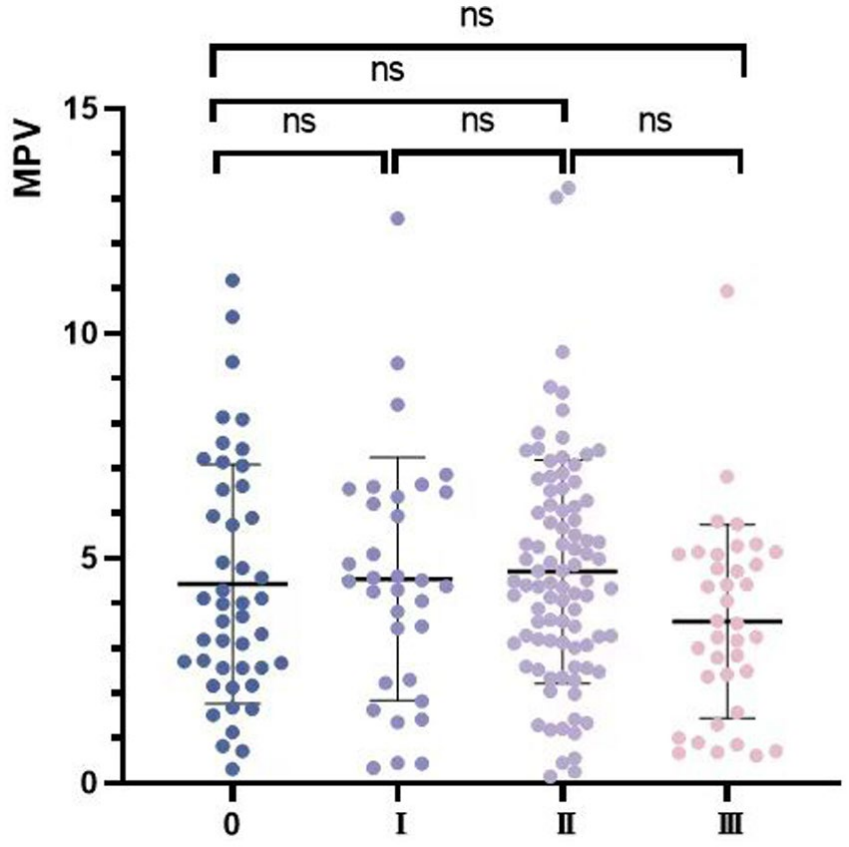
The scatter plot shows the variation of mastoid pneumatization volume in different groups. MPV, mastoid pneumatization volume.

There was no significant difference in mastoid air cell volumes between different sides (*p* = 0.654), but the volume was smaller in female (3.83 ± 2.03 cm^3^) than male group (5.87 ± 3.01 cm^3^, *p* ≤ 0.001), which consistent with previous reports (16, 17). The mastoid air cell volumes of Grade I, II, and III were 4.46 ± 2.62 cm^3^, 4.70 ± 2.49 cm^3^, and 3.59 ± 2.15 cm^3^, respectively. There was no significantly correlation between HJBs and mastoid air cell volume in all the patients (*R*^2^ = 0.004, *p* = 0.165, Table 1 and Figure 3). While the same result was found in the unilateral HJB population, no significantly difference in mastoid air cell volumes between the HJB and normal side was found (*p* = 0.736).

In both ears, Grade II was most common. As in other studies, we also found a predilection for the right ear, this is thought to result from the asymmetry of the embryonic vena cava (18). Of the 79 left HJB cases, 30 were in Grade I (30/79, 38.0%), 36 in Grade II (36/79, 45.6%), and 13 in Grade III (13/79, 16.4%). Of the 102 right HJB cases, 24 were in Grade I (24/102, 23.5%), 54 in Grade II (54/102, 53.0%), and 24 in Grade III (24/102, 23.5%). Of the total 181 HJB cases, 54 were in Grade I (54/181, 29.8%), 90 in Grade II (90/181, 49.7%), and 37 in Grade III (37/181, 20.5%; Table 2). The chi-square test result shows that HJBs were more biased to the right side (56.4% vs. 43.6%, *p* ≤ 0.001).

**Table 2:** Comparison of the mastoid volume with different degree of high jugular bulb in two sides.

	Left		Right		Number
No HJB	34	4.17 ± 2.15	11	5.23 ± 3.85	45
I	30	4.54 ± 2.78	24	4.36 ± 2.47	54
II	36	4.89 ± 2.61	54	4.58 ± 2.42	90
III	13	4.06 ± 2.80	24	3.34 ± 1.72	37
*p*-value		0.620		0.127	

## Discussion

The junction of the sigmoid sinus and the internal jugular vein is called the JB. The sigmoid sinus joins the jugular vein at the JB. It is a slightly enlarged segment of the internal jugular vein that locates within a circular cavity of bony jugular foramen just below the posterior part of the middle ear floor. The JB is a dynamic structure that is not stable until adulthood (19). A common vascular variant of JB is HJB. It is often defined as the dome of the JB above the inferior bony annulus, the inferior edge of the internal acoustic meatus (IAM) or the basal turn of the cochlea. HJB is often discovered as an asymptomatic detection. Pulsatile venous tinnitus, vertigo, vestibular dysfunction and hearing disorders can be observed in patients with a HJB (20–24).

Vestibular dysfunction is usually associated with damage to the internal auditory canal, endolymphatic duct or posterior semicircular canals (4, 22). Abnormalities of the vestibular aqueduct (VA) and the JB have been suggested as factors of eroding into inner ear structures. The jugular bulb related vestibular aqueduct dehiscence is the most common JB-related inner ear dehiscence, which has been associated with Meniere disease or endolymphatic hydrops. It can be postulated that the interruption of VA may occur when the HJB reaches to a sufficient height, such as above the lower edge of the IAC, which pathological feature of VA related HJB (25, 26). Conductive hearing loss is caused by the JB touching the tympanic membrane or extending to a small portion of the ossicular chain and round window. A HJB is susceptible to surgical procedures such as myringotomy or other interventions (7, 20, 27–29). Especially in the case of lateral skull base surgery, the surgeon must be able to handle it as it can obstruct a clear operative view. Physicians should pay attention to the variation of HJB to avoid complications from unexpected massive bleeding.

The relationship between HJB and temporal bone pneumatization has been controversial. Graham MD (22) proposed that the height and shape of the JB are related to pneumatization of the temporal bone based on histopathologic specimens. Similarly, this hypothesis was also supported by Wadin et al. (30) and Kennedy et al. (31). They report that HJB occurs mainly in low-grade mastoid bone pneumatization. However, Orr et al. (8) showed the opposite conclusion that JB position and shape had nothing to do with temporal bone pneumatization. The research method used to elucidate the correlation in this study differs from that of previous researchers, as we used an innovative approach to investigate quantitative differences in air cell volumes in mastoid of different HJB type. As a result, no significant difference of mastoid air cell volume between the severe HJB group and the mild HJB group was observed. This result is also consistent with the study of Orr JB.

Our study found that there was no correlation between the severity of HJBs and the mastoid pneumatization in patients. In the selected HJB group, the mastoid air cell volume was smaller in the female group, suggesting that they may be prone to chronic otitis media. According to the genetic theory, Minimal pneumatization of the temporal bone is characteristic of otitis media (32, 33). In the selected HJB group, the mastoid air cell volume was smaller in the female group, which consistented with previous reports (16, 17). As a non-invasive imaging examination, the significance of HRCT can be extended to multi-center cross-sectional studies with large samples, greatly improving its reliability. Future investigations will require a larger number of radiological imaging’s.

## Conclusion

In this study, we state a developmental relationship between HJB and mastoid air cells. No developmental relationship between the mastoid pneumatization and the degree of the HJBs was observed. In addition, HJB mostly occurs on the right side. Atilla et al. (21), Aksoy et al. (34) and Wang et al. (35) measured previously that the HJB was asymmetric and right-sided dominant in normal conditions, which was consistent with our findings. This was thought to be caused by asymmetry of the embryonic vena cava (19). Further studies, preferably repeated in larger statistical populations, may be needed to clarify the above findings.

## Data availability statement

The raw data supporting the conclusions of this article will be made available by the authors, without undue reservation.

## Ethics statement

The studies involving humans were approved by the First Affiliated Hospital of Fujian Medical University. The studies were conducted in accordance with the local legislation and institutional requirements. Written informed consent for participation was not required from the participants or the participants’ legal guardians/next of kin in accordance with the national legislation and institutional requirements.

## Author contributions

CC: Conceptualization, Data curation, Formal analysis, Investigation, Methodology, Project administration, Software, Visualization, Writing–original draft, Writing–review and editing. SW: Data curation, Software, Visualization, Writing–original draft. ZC: Visualization, Writing–original draft, Writing–review and editing. YC: Writing–review and editing. GY: Writing–review and editing. XH: Writing–review and editing. XG: Resources, Supervision, Writing–review and editing. CL: Funding acquisition, Resources, Supervision, Writing–review and editing.

## Footnotes

^1^ http://rsbweb.nih.gov/ij/

## References

[1] Cros O Knutsson H Andersson M Pawels E Borga M Gaihede M. Determination of the mastoid surface area and volume based on micro-CT scanning of human temporal bones. Geometrical parameters depend on scanning resolutions. Hear Res. (2016) 340:127–34. doi: 10.1016/j.heares.2015.12.005, PMID: 26701785

[2] Mey KH Sørensen MS Homøe P. Histomorphometric estimation of air cell development in experimental otitis media. Laryngoscope. (2006) 116:1820–3. doi: 10.1097/01.mlg.0000233540.26519.ba, PMID: 17003720

[3] Friedmann DR Eubig J Winata LS Pramanik BK Merchant SN Lalwani AK. Prevalence of jugular bulb abnormalities and resultant inner ear dehiscence: a histopathologic and radiologic study. Otolaryngol Head Neck Surg. (2012) 147:750–6. doi: 10.1177/0194599812448615, PMID: 22619257

[4] Friedmann DR Le BT Pramanik BK Lalwani AK. Clinical spectrum of patients with erosion of the inner ear by jugular bulb abnormalities. Laryngoscope. (2010) 120:365–72. doi: 10.1002/lary.20699, PMID: 19924772

[5] Friedmann DR Eubig J Winata LS Pramanik BK Merchant SN Lalwani AK. A clinical and histopathologic study of jugular bulb abnormalities. Arch Otolaryngol Head Neck Surg. (2012) 138:66–71. doi: 10.1001/archoto.2011.231, PMID: 22249632

[6] Aladeyelu OS Olojede SO Lawal SK Mbatha WE Sibiya AL Rennie CO. Influence of pneumatization on morphology of temporal bone-related vasculatures and their morphometric relationship with ear regions: a computed tomography study. Sci Rep. (2023) 13:1996. doi: 10.1038/s41598-023-29295-4, PMID: 36737493 PMC9898243

[7] Graham MD. The jugular bulb: its anatomic and clinical considerations in contemporary otology. Laryngoscope. (1977) 87:105–25. doi: 10.1288/00005537-197701000-00013, PMID: 187882

[8] Orr JB Todd NW. Jugular bulb position and shape are unrelated to temporal bone pneumatization. Laryngoscope. (1988) 98:136–8. doi: 10.1288/00005537-198802000-00003, PMID: 3339920

[9] Dai PD Zhang HQ Wang ZM Sha Y Wang KQ Zhang TY. Morphological and positional relationships between the sigmoid sinus and the jugular bulb. Surg Radiol Anat. (2007) 29:643–51. doi: 10.1007/s00276-007-0266-5, PMID: 17962901

[10] Luntz M Malatskey S Tan M Bar-Meir E Ruimi D. Volume of mastoid pneumatization: three-dimensional reconstruction with ultrahigh-resolution computed tomography. Ann Otol Rhinol Laryngol. (2001) 110:486–90. doi: 10.1177/000348940111000516, PMID: 11372935

[11] Cinamon U. The growth rate and size of the mastoid air cell system and mastoid bone: a review and reference. Eur Arch Otorhinolaryngol. (2009) 266:781–6. doi: 10.1007/s00405-009-0941-8, PMID: 19283403

[12] Alper CM Kitsko DJ Swarts JD Martin B Yuksel S Cullen Doyle BM. Role of the mastoid in middle ear pressure regulation. Laryngoscope. (2011) 121:404–8. doi: 10.1002/lary.21275, PMID: 21271597 PMC3037018

[13] Dai P Zhang T Wang K Song J Qian W Wang Z. Positional relationship between the facial nerve and other structures of the temporal bone. J Laryngol Otol. (2004) 118:106–11. doi: 10.1258/002221504772784540, PMID: 14979946

[14] Woo CK Wie CE Park SH Kong SK Lee IW Goh EK. Radiologic analysis of high jugular bulb by computed tomography. Otol Neurotol. (2012) 33:1283–7. doi: 10.1097/MAO.0b013e318259b6e7, PMID: 22722144

[15] Chen C Huang X Chen Z Liu Y Chen Z Zeng C. The age-related growth of mastoid air cells in infancy: a retrospective cross-sectional study. Otol Neurotol. (2023) 44:e583–7. doi: 10.1097/MAO.0000000000003931, PMID: 37442589

[16] Aladeyelu OS Olaniyi KS Olojede SO Mbatha WE Sibiya AL Rennie CO. Temporal bone pneumatization: a scoping review on the growth and size of mastoid air cell system with age. PLoS One. (2022) 17:e0269360. doi: 10.1371/journal.pone.0269360, PMID: 35657972 PMC9165849

[17] Sasani H Etli Y Tastekin B Hekimoglu Y Keskin S Asirdizer M. Sex estimation from measurements of the mastoid triangle and volume of the mastoid air cell system using classical and machine learning methods: a comparative analysis. Am J Forensic Med Pathol. (2023). doi: 10.1097/PAF.0000000000000890 [epubh ahead of print]., PMID: 38039501

[18] Kawano H Tono T Schachern PA Paparella MM Komune S. Petrous high jugular bulb: a histological study. Am J Otolaryngol. (2000) 21:161–8. doi: 10.1016/S0196-0709(00)85018-8, PMID: 10834549

[19] Friedmann DR Eubig J McGill M Babb JS Pramanik BK Lalwani AK. Development of the jugular bulb: a radiologic study. Otol Neurotol. (2011) 32:1389–95. doi: 10.1097/MAO.0b013e31822e5b8d 21921860

[20] Ball M Elloy M Vaidhyanath R Pau H. Beware the silent presentation of a high and dehiscent jugular bulb in the external ear canal. J Laryngol Otol. (2010) 124:790–2. doi: 10.1017/S0022215109992349, PMID: 20025812

[21] Atilla S Akpek S Uslu S Ilgit ET Işik S. Computed tomographic evaluation of surgically significant vascular variations related with the temporal bone. Eur J Radiol. (1995) 20:52–6. doi: 10.1016/0720-048X(95)00619-2, PMID: 7556255

[22] Sayit AT Gunbey HP Fethallah B Gunbey E Karabulut E. Radiological and audiometric evaluation of high jugular bulb and dehiscent high jugular bulb. J Laryngol Otol. (2016) 130:1059–63. doi: 10.1017/S0022215116009166, PMID: 27823580

[23] Oztürkcan S Katilmiş H Ozkul Y Erdoğan N Başoğlu S Tayfun MA. Surgical treatment of the high jugular bulb by compressing sinus sigmoideus: two cases. Eur Arch Otorhinolaryngol. (2008) 265:987–91. doi: 10.1007/s00405-007-0545-0, PMID: 18046566

[24] Kieran SM Meyer TA. Cochlear Ménière’s disease in association with a high jugular bulb. Otol Neurotol. (2015) 36:e146–7. doi: 10.1097/MAO.0000000000000502, PMID: 25118573

[25] Noyalet L Ilgen L Bürklein M Shehata-Dieler W Taeger J Hagen R. Vestibular aqueduct morphology and Meniere’s disease-development of the “vestibular aqueduct score” by 3D analysis. Front Surg. (2022) 9:747517. doi: 10.3389/fsurg.2022.747517, PMID: 35187054 PMC8854222

[26] Bächinger D Luu NN Kempfle JS Barber S Zürrer D Lee DJ. Vestibular aqueduct morphology correlates with endolymphatic sac pathologies in Menière’s disease-a correlative histology and computed tomography study. Otol Neurotol. (2019) 40:e548–55. doi: 10.1097/MAO.0000000000002198, PMID: 31083097 PMC6554006

[27] Fox R Nash R Tatla T. Encountering a high jugular bulb during ear surgery. Ann R Coll Surg Engl. (2017) 99:36–7. doi: 10.1308/rcsann.2016.0290, PMID: 27659376 PMC5392809

[28] Vachata P Petrovicky P Sames M. An anatomical and radiological study of the high jugular bulb on high-resolution CT scans and alcohol-fixed skulls of adults. J Clin Neurosci. (2010) 17:473–8. doi: 10.1016/j.jocn.2009.07.121, PMID: 20167495

[29] Wang CH Shi ZP Liu DW Wang HW Huang BR Chen HC. High computed tomographic correlations between carotid canal dehiscence and high jugular bulb in the middle ear. Audiol Neurootol. (2011) 16:106–12. doi: 10.1159/000314755, PMID: 20606423

[30] Wadin K Wilbrand H. The topographic relations of the high jugular fossa to the inner ear. A radioanatomic investigation. Acta Radiol Diagn. (1986) 27:315–24. doi: 10.1177/028418518602700312, PMID: 3751681

[31] Kennedy DW El-Sirsy HH Nager GT. The jugular bulb in otologic surgery: anatomic, clinical, and surgical considerations. Otolaryngol Head Neck Surg. (1986) 94:6–15. doi: 10.1177/019459988609400102, PMID: 3081858

[32] Todd NW Pitts RB Braun IF Heindel H. Mastoid size determined with lateral radiographs and computerized tomography. Acta Otolaryngol. (1987) 103:226–31. doi: 10.3109/00016488709107277, PMID: 3577754

[33] Turgut S Tos M. Correlation between temporal bone pneumatization, location of lateral sinus and length of the mastoid process. J Laryngol Otol. (1992) 106:485–9. doi: 10.1017/S0022215100119942, PMID: 1624879

[34] Aksoy SH Yurdaisik I. Co-occurrence of vertebral artery hypoplasia and high jugular bulb in patients undergoing cranial magnetic resonance imaging. Acta Neurol Belg. (2022) 122:369–75. doi: 10.1007/s13760-021-01619-z, PMID: 33569702

[35] Wang J Feng Y Wang H Li C Wu Y Shi H. Prevalence of high jugular bulb across different stages of adulthood in a Chinese population. Aging Dis. (2020) 11:770–6. doi: 10.14336/AD.2020.0215, PMID: 32765944 PMC7390519

